# Editorial Note: The Communication Factor EDF and the Toxin–Antitoxin Module *mazEF* Determine the Mode of Action of Antibiotics

**DOI:** 10.1371/journal.pbio.3002325

**Published:** 2023-09-18

**Authors:** The PLOS Biology Editors

Readers have raised concerns about quantitative results published in bar graphs in Figures S1, S2, S4, and S5 of this article [1, 2]. Specifically, it was noted that the bars are not consistently spaced within the bar graphs; some data annotations (values) on the graphs do not agree with bars’ alignment with values on the Y axis; and some bars have lower edges that appear slightly above or below the X axis line.

The authors stand by the results and commented that these issues are due to the method(s) used to prepare the graphs. They stated that they used an early illustrator program into which they copied the values of calculated results (means and standard deviations) of three independent experiments for plotting on electronic millimeter grid paper [3].

The authors also stated that in instances where data annotations within the graphs differ from bars’ alignment with the Y axis, the data labels within the graphs (i.e., above the bars) provide the correct results.

The authors stated that the original raw data underlying the graphs of concern are not fully available at this time given the time elapsed. In light of this issue, PLOS has been unable to verify the results and the article does not currently comply in full with the applicable version of the PLOS Data Availability policy*. Nevertheless, the editors consider that the explanation provided by the authors sufficiently clarifies how the graphs were generated.

*The PLOS Data Availability policy was updated in 2014. *The former policy (which was in place in 2008–09, [4]) still required authors to make data integral to an article’s findings freely available without restriction*. *The pre-2014 policy advised that data should be provided in supporting information files, a public repository, or upon request, but it did not strictly require publication or deposition of all quantitative data*.
